# Dual Drug Delivery in Cochlear Implants: In Vivo Study of Dexamethasone Combined with Diclofenac or Immunophilin Inhibitor MM284 in Guinea Pigs

**DOI:** 10.3390/pharmaceutics15030726

**Published:** 2023-02-22

**Authors:** Wiebke Behrends, Katharina Wulf, Stefan Raggl, Max Fröhlich, Thomas Eickner, Dana Dohr, Karl-Heinz Esser, Thomas Lenarz, Verena Scheper, Gerrit Paasche

**Affiliations:** 1 Department of Otolaryngology, Hannover Medical School, 30625 Hannover, Germany; 2 Auditory Neuroethology and Neurobiology, Institute of Zoology, University of Veterinary Medicine Hannover Foundation, 30559 Hannover, Germany; 3 Institute for Biomedical Engineering, Rostock University Medical Center, 18119 Rostock, Germany; 4 MED-EL Medical Electronics, 6020 Innsbruck, Austria; 5 MED-EL Research Center, 30625 Hannover, Germany; 6 Department of Otorhinolaryngology, Head and Neck Surgery “Otto Körner”, Rostock University Medical Center, 18057 Rostock, Germany; 7 Hearing4all Cluster of Excellence, Hannover Medical School, 30625 Hannover, Germany

**Keywords:** dual drug delivery, cochlear implants, diclofenac, immunophilin inhibitor MM284, polymeric coating

## Abstract

Cochlear implants are well established to treat severe hearing impairments. Despite many different approaches to reduce the formation of connective tissue after electrode insertion and to keep electrical impedances low, results are not yet satisfying. Therefore, the aim of the current study was to combine the incorporation of 5% dexamethasone in the silicone body of the electrode array with an additional polymeric coating releasing diclofenac or the immunophilin inhibitor MM284, some anti-inflammatory substances not yet tested in the inner ear. Guinea pigs were implanted for four weeks and hearing thresholds were determined before implantation and after the observation time. Impedances were monitored over time and, finally, connective tissue and the survival of spiral ganglion neurons (SGNs) were quantified. Impedances increased in all groups to a similar extent but this increase was delayed in the groups with an additional release of diclofenac or MM284. Using Poly-L-lactide (PLLA)-coated electrodes, the damage caused during insertion was much higher than without the coating. Only in these groups, connective tissue could extend to the apex of the cochlea. Despite this, numbers of SGNs were only reduced in PLLA and PLLA plus diclofenac groups. Even though the polymeric coating was not flexible enough, MM284 seems to especially have potential for further evaluation in connection with cochlear implantation.

## 1. Introduction

Today, cochlear implant (CI) technology is the standard procedure for the treatment of children born deaf or of adults who become deaf during their lives. Therefore, the CI undisputedly represents a major clinical success. The indication also holds great potential for increasingly older people to maintain and improve their quality of life with hearing prostheses. Nevertheless, the results vary from patient to patient. Some patients have no or only very limited improvement in hearing despite a CI. One possible reason for this is the interface between the electrode array and the auditory nerve. The CI uses electrical pulses to stimulate the auditory nerve via an electrode. After implantation of the electrode in the cochlea, connective tissue forms around the electrode array, ranging from a few cells to new bone formation [1]. This connective tissue impairs signal transmission to the neurons of the auditory nerve, leading to unpredictable stimulus propagation in the cochlea and, thus, to poorer frequency selectivity, which is contrary to a hearing impression that should be as natural as possible for the patient. Measurements of these changes can be obtained by observing changes in impedances [2].

Impedances increase sharply in the first days after implantation; after about three weeks, they remain stable, and a plateau is formed [3]. Thus, connective tissue growth should be complete by this time. It is possible to influence the growth of connective tissue by using anti-inflammatory substances [4]. Less tissue growth should be reflected in impedance measurements in the form of lower impedances compared to untreated groups.

In clinical practice, medications can be applied systemically [5] and locally [6] into the inner ear in the context of cochlear implantations. These are mostly anti-inflammatory glucocorticoids such as dexamethasone (DEX), prednisolute, or triamcinolone. Other drug delivery systems take advantage of the permeability of the round window membrane by applying a drug-carrying hydrogel to the round window membrane via the middle ear [7]. The gel releases the drug and allows it to diffuse through the round window membrane into the inner ear [8]. However, when administered via the round window membrane, the distribution of substances in the cochlea is very limited [9].

Several approaches are being explored for direct delivery of compounds into the cochlea [10]. These include pump-based drug delivery [11] as well as incorporation of substances into implant coatings [12,13] or colonization of the implant surface with drug-releasing cells [14]. Most of these approaches aim to preserve cochlear neurons (spiral ganglion neurons, SGNs) through the administration of neurotrophic factors. Others, such as the use of pumps, carry an additional infection risk due to the need for permanent access to the cochlea [15].

In other areas of implant technology, the coating of implant surfaces with drug-containing polymers is already a proven concept for targeted control of implant–tissue interaction. For example, stents are coated with drug-containing polymeric coatings to prevent in-stent restenosis by controlled, long-lasting drug release [16]. Initial tests have also been carried out on CIs with regard to drug coating. To control and adjust the drug release depending on the site of action, different modifications of the matrix or coating systems can be used [17]. Particular attention was paid to possible carriers for drugs as coatings for silicone bodies. Poly-L-lactides (PLLAs) have been shown to have no negative effects on the survival of inner ear neurons compared to pure silicone bodies [18]. In addition, it was reported that a PLLA-coated electrode shows no difference in the generated pressure and friction during insertion compared to an uncoated electrode at higher insertion speeds [19]. In contrast, possible effects on connective tissue growth have not yet been investigated.

Different substances have inhibitory effects on anti-inflammatory processes. Diclofenac is a drug already approved in human medicine, which is mainly used for musculoskeletal problems. It is a COX-1 and COX-2 inhibitor and has antiphlogistic, analgesic, and antipyretic effects [20]. Thus far, it has not been used in the inner ear.

In in vitro experiments, diclofenac was shown to be compatible with the SGNs, making it a candidate for use in the inner ear [21]. A reducing effect on connective tissue growth can be expected [22]. Similarly, the immunophilin inhibitor MM284, which is a cyclosporine derivate that reduces the recruitment of T cells and macrophages and leads to a reduction in inflammatory processes without affecting the immune system, appeared suitable for application to the ear at concentrations of or below 4 × 10^−5^ mol/L [23]. 

Both compounds were tested for their tolerability (SGN survival) and efficacy (reduction in connective tissue growth) when released from a PLLA coating on CIs in vivo in this study. Each electrode body contained 5% DEX mixed into the silicone. Previous studies have demonstrated that DEX has anti-inflammatory and immune suppressive functions [24] and is not toxic for cultured SGNs in concentrations up to 1 × 10^−4^ mol/L [13]. There are already many studies in which DEX has been incorporated in the silicone of the CI electrode in different concentrations [13,25]. Despite the positive results of previous studies [26], impedances continued to increase significantly over a 28-day period compared to day 0 after CI implantation when electrode arrays with 1% and 10% DEX were used [2]. After 28 days, impedances reached a plateau. To reduce the increase in impedances during the first 28 days, we tested whether a combination of DEX with modified surfaces provides better results in terms of connective tissue reduction. Electrodes with DEX in the silicone body were examined. Electrodes were additionally provided with a PLLA coating, which contained either diclofenac or MM284. The performance of the modified electrodes was tested in vivo regarding impedances, tissue growth, and SGN density.

## 2. Materials and Methods

### 2.1. Ethical Statement

The in vivo experiments in this study were conducted in accordance with the German Animal Welfare Law and the European Directive 2010/63 and approved by the State Office for Consumer Protection and Food Safety, Dept. of Animal Welfare under the number 20/3502. Guinea pigs were housed in the animal facility of the Lower Saxony Center for Biomedical Engineering, Implant Research and Development (NIFE) of Hannover Medical School, and the experiments were performed with regard to the valid directives regarding accommodation, care, and usage of experimental animals. The guinea pigs were maintained in a temperature- and humidity-controlled room, exposed to a 24 h light–dark cycle (14 h/10 h) with free access to food and water.

### 2.2. Electrode Arrays

**Figure 1:**
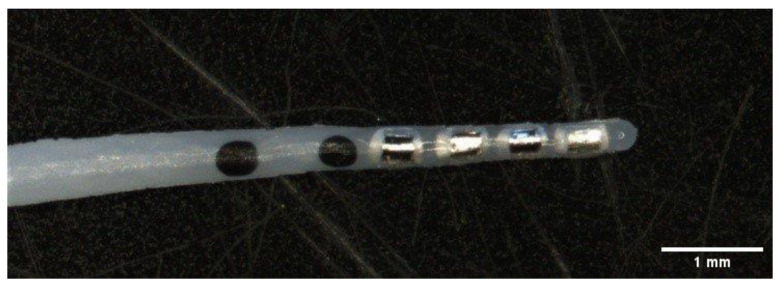
Electrode array with 5% DEX in the silicone. Four contacts and two marker dots at 3 and 4 mm from the tip, respectively.

Cochlear implant guinea pig electrode arrays (MED-EL GmbH, Innsbruck, Austria) comprised four platinum (Pt) contacts at the distal tip and an additional reference electrode. Furthermore, the connector had an attached mesh for better fixation of the setup to the guinea pig skull. Electrode insertion depth was controlled with two black marker dots at 3 and 4 mm. The diameter of the electrode at the tip was approximately 300 µm (Figure 1). For each electrode, 5% DEX (Sanofi, Paris, France) was incorporated into the silicone. A shielded cable connected the electrode array to a MED-EL PULSAR CI100 cochlear implant, which, in turn, was inductively coupled via a coil to the MED-EL MAX-Box.

### 2.3. Coating of the Electrodes

The coating of the electrodes from the tip to the marker dot at 4 mm was performed according to Wulf et al. 2022 [21]. In order to achieve a stable PLLA coating, the electrode arrays were rinsed three times with ethanol and subsequently activated via O_2_-plasma using a 100 W power at 0.3 mbar for 1 min in a plasma chamber (Diener, Ebhausen, Germany). Then, the samples were incubated in pure (3-glycidyloxypropyl) trimethoxysilane (GOPS) (Merck, Darmstadt, Germany) for 4 h at 90 °C. The activated samples were rinsed three times with ethanol and dried at 80 °C overnight under vacuum at 40 mbar. The polymer coating of the electrode array was prepared via an established and characterized in-house-manufactured spray coating process. First, the activated electrodes were spray coated with a thin polymer layer of PLLA-NH_2_ (VWR, Dresden, Germany) using a chloroform PLLA-NH_2_ (2 wt%) spray solution. Afterward, the samples were dried at 80 °C overnight and coated with chloroform PLLA (0.2 wt%) spray solutions containing either pure PLLA Resomer L210 (Evonik, Schwerte, Germany) or a polymer/drug mixture. These mixtures contained either diclofenac sodium salt (Merck, Darmstadt, Germany) to PLLA at ratios of 20:80 wt% or the immunophilin inhibitor MM284 (G. Fischer, MPIBC, Halle/S., Germany) to PLLA at a ratio 10:90 wt% in order to reach layer thicknesses of about 10 µm corresponding to a 70 µg coating mass. Thus, drug contents resulted in 14 µg diclofenac and 7 µg MM284 per electrode, respectively. Afterward, the modified electrodes were dried at 80 °C overnight. As the four electrode contacts were masked during the manufacturing process of the electrode, the coating was removed from the contacts by removing the masked areas according to [21]. Electrodes were sterilized using ethylene oxide.

### 2.4. Animal Groups

Four groups of six animals were formed (Table 1). Each animal received a CI unilaterally in the left inner ear. The right side remained untreated and served as a reference.

### 2.5. Animals and Experimental Design

Twenty-four adult male Dunkin Hartley guinea pigs (Charles River Laboratories, Châtillon, France) weighing 375 to 540 g were used. All animals were normal hearing as proven by initial hearing threshold determination (see Section 2.7, AABR). After hearing threshold determination on day 0, the CI electrode was implanted. Subsequently, impedances were measured on all four contacts. From day 1 to 14, impedances were measured daily on the awake animal. After day 14, impedances were measured weekly on days 21 and 28. On the final day (day 28), an additional hearing threshold determination was performed under anesthesia and perilymph was collected from the apex of the cochleae as described in [27]. Still under general anesthesia, the animals were euthanized immediately through transcardial perfusion with 200 mL of phosphate-buffered saline (PBS, Gibco™, Thermo Fisher Scientific, Paisley, UK) followed by 100 mL of 4% paraformaldehyde (PFA, Merck KGAA, Darmstadt, Germany). After perfusion, animals were decapitated and temporal bones were removed and dissected under a dissection microscope to expose the cochleae. The apex and oval window were pierced to enable rinsing. The electrode arrays remained in situ.

### 2.6. Anesthesia, Medication, and Surgery

**Figure 2:**
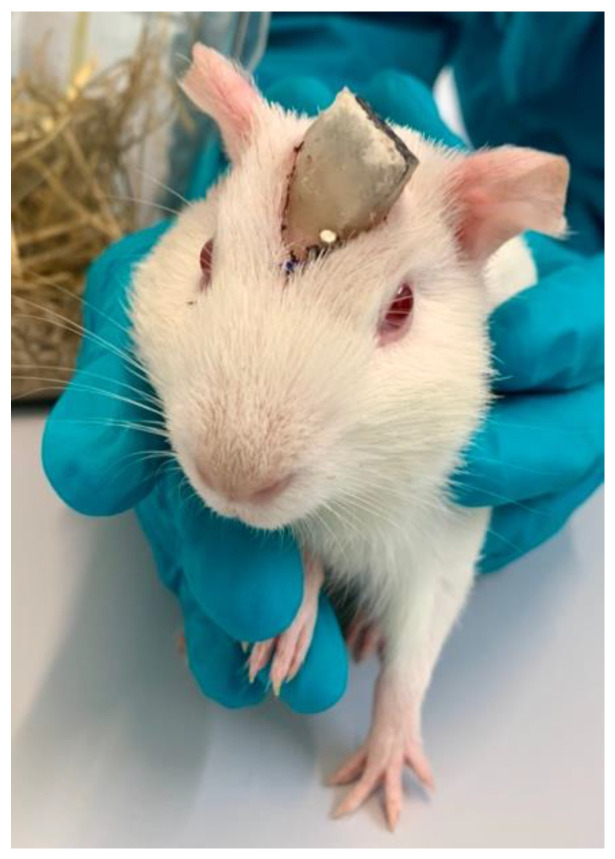
Guinea pig with connector attachment.

Anesthesia and medication after surgery were previously published in detail by Malfeld et al. [28]. The implantation was performed under general anesthesia (intramuscular medetomidine hydrochloride 0.2 mg/kg, midazolam 1 mg/kg, and fentanyl 0.025 mg/kg) with previous sedation (oral diazepam 4 mg/kg). In addition, the animals received Bepanthen^®^ eye ointment to avoid eye desiccation, and analgesia (Meloxicam 0.2 mg/kg s.c.) and antibiotics (Enrofloxacin 10 mg/kg s.c.) to reduce pain and to prevent infections, respectively. After anesthetizing and determination of the hearing threshold (see Section 2.7 AABR), animals were placed on a heating mat at 38 °C. On day 0, after hearing threshold determination, all animals were implanted unilaterally in the left inner ear via cochleostomy and using an electrode insertion trauma approach [2]. Areas of the skin to be incised were locally infiltrated with prilocaine (Xylonest 1%, Aspen Germany GmbH, Munich, Germany). On the guinea pig skull, the skin was opened and the top of the skull was dissected. The electrode and reference electrode were then guided underneath the skin and muscles toward the *bulla tympanica* using a tunnel tube. Via an approach behind the ear, the periosteum of the bulla was abscised and the middle ear was opened with a scalpel and a forceps. A 0.7 mm hole was drilled (AccuPen 6V+; RISystem AG, Landquart, CH) in the cochlea 1 mm below the round window, into which the electrode was subsequently inserted up to the second black marker dot (Figure 1) resulting in 4 mm insertion of the active electrode array. The electrode was fixed to the edge of the bulla using dental UV-cement (Tetric EvoFlow^®^, Ivoclar Vivadent, Ellwangen, Germany) and the hole in the bulla was sealed. The reference electrode was placed above the bulla defect in the musculature. Then, the postauricular wound was closed in two layers. With the aid of two screws anchored in the skull and using Tetric EvoFlow and methyl methacrylate (Paladur^®^, Kulzer GmbH, Hanau, Germany), the connector was fixed to the skull (Figure 2). The UV-cement was used as a foundation and the Paladur was applied generously around the connector. Thereafter, the anesthesia was antagonized with Naloxon (0.03 mg/kg s.c., Naloxon, Inresa Arzneimittel GmbH, Freiburg, Germany), Flumazenil (0.1 mg/kg s.c., Flumazenil, Hameln pharma GmbH, Hameln, Germany), and Atipamezol (1.0 mg/kg s.c., Atipazole, Prodivet pharmaceuticals sa/nv, Eynatten, Belgium). Following antagonization, the guinea pig was placed under red light until it could maintain its body temperature, but at least for one hour. 

### 2.7. Acoustically Evoked Auditory Brainstem Response (AABR)

The AABR measurements were performed under general anesthesia on day 0 and day 28 in a sound attenuating chamber using a TDT System (Tucker-Davis Technologies, Alachua, FL, USA), and data were analyzed using the corresponding BioSigRP software. Acoustic tone stimuli with frequencies of 1, 2, 4, 8, 16, 32, and 40 kHz were generated and presented at sound pressure levels (SPLs) from 100 dB down to 0 dB in 5 dB steps by an EC1 speaker (Tucker-Davis Technologies) positioned in the external ear canal. One speaker was used first for the left ear and subsequently for the right ear. Subdermal needle electrodes (CareFusion Nicolet, Middleton, WI, USA) were placed at the vertex (common positive), left and right mastoid (references), and in the neck (ground). The tone pulses had a duration of 10 ms with a square cosine rise and fall time of 1 ms. The repetition rate was 10 tones per second and the recorded signal was bandpass-filtered from 300 to 3000 Hz to suppress the inclusion of background noise. All recorded neurological signals were sampled 300 times and averaged for analysis. Thresholds were defined as the lowest stimulus required to evoke a visually replicable waveform. When no thresholds could be detected, a value of 100 dB was used for calculation of mean values and threshold shifts.

### 2.8. Impedance Measurements

Impedance measurements were performed on days 0 and 28 in the anesthetized animal and on all other days on the awake animal. For the impedance measurements, the clinical system from MED-EL was used consisting of a MAX-Box and the MAESTRO software (version 8.0). Electrode impedances were measured via the Impedance Field Telemetry (IFT) task in monopolar mode.

### 2.9. Histology

#### 2.9.1. Tissue Preparation

Histologic treatment of the cochleae was adapted from MacDonald and Rubel 2008 [29]. All preparation steps were performed at room temperature on a platform rotator. The dissected cochleae were post-fixed in PFA overnight and rinsed in PBS for 10 min three times the next day. They were then decalcified in 10% ethylenediaminetetraacetic acid disodium salt (EDTA, Sigma-Aldrich Chemie GmbH, Schnelldorf, Germany) in PBS, pH 7.4, for three to four weeks, with regular EDTA change every one to three days. After decalcification, the cochleae were immunohistochemically stained. 

To prepare staining, the cochleae were stored in 1% Triton X-100 (Sigma-Aldrich) in PBS at room temperature for six to eight hours. The primary antibody (Anti-vimentin antibody produced in goat, Sigma-Aldrich Chemie GmbH) was diluted in blocking solution (5% NHS (normal horse serum, Biozol, Eching, Germany) and 1% Triton in PBS). It was then applied to the cochleae and incubated for three days at 4 °C. After the incubation, the samples were washed with PBS three times for 2 h each. The second antibody (Alexa Fluor 647-conjugated AffiniPure Bovine Anti-Goat igG, Jackson ImmunoResearch, Pennsylvania) was applied, incubated, and washed in the same way. As a next step, the cochleae were dehydrated (70% ethanol overnight, 95% ethanol for 30 min, and ethanol absolute for 2 h on a rotation platform). The last step was the refractive index adjustment. For this, MSBB (methyl salicylate benzyl benzoate) was made by mixing five parts methyl salicylate with three parts benzyl benzoate (Merck KGAA). The MSBB was mixed 1:1 with ethanol absolute and the cochlea was placed in it for four hours at room temperature on a rotation platform. The cochlea was placed in pure MSBB overnight at room temperature and then stored at 4 °C.

#### 2.9.2. Imaging

For microscopy, a Leica SP8 laser scanning confocal microscope (Leica Microsystems GmbH, Wetzlar, Germany) equipped with a white light laser was used combined with the Leica Confocal Software (LAS X Science Microscope Software; version LAS X 3.5.7.23225). An objective lens with 10× magnification (HC PL Fluotar 10x/0.30 Dry, Fa. Leica) was used. The laser provided the excitation lines at 492 nm (for PFA-induced autofluorescence) and 652 nm (for Alexa Fluor 647). The slices were generated with 20 µm steps (z-stack on) at a scanning speed of 400 Hz, 5× line averaging, and 3× frame averaging. To be able to see the electrode, the TLD (brightfield detector/through the lens detector) was switched on as third channel.

#### 2.9.3. Connective Tissue Quantification

To assess the tissue response in each turn of the cochlea, the Leica Confocal software (LAS X Science Microscope Software) was used. With two custom-made ranking scores (adapted from [30]), the connective tissue growth was evaluated. One subjective scoring for all 7 cross-sections of the cochlea (lower basal turn (lb), upper basal turn (ub), first middle turn (1.m), second middle turn (2.m), third middle turn (3.m), fourth middle turn (4.m), and apical turn (a)) was defined as described in Table 2 applying the percentage of filling of the respective cross-sectional scala tympani area.

A second ranking score for the connective tissue growth around the single contacts of the electrode array was defined: score 0 = no connective tissue; score 1 = thin film of tissue directly on the contact; score 2 = reticular growth of connective tissue on the contact; score 3 = contact completely covered by compact connective tissue. 

#### 2.9.4. Spiral Ganglion Neuron Counting

Neurons were automatically counted in five midmodiolar slices per cochlear segment using the ITCN plug-in (Image-based Tool for Counting Nuclei, Center for Bio-Image Informatics, http://www.bioimage.ucsb.edu/downloads/automatic-nuclei-counter-plug-in-for-imagej, accessed on 10 May 2022) for ImageJ software (Wayne Rasband, National Institutes of Health, Bethesda, MD, USA). 

In the sections, the circumferences of Rosenthal’s canal were measured at the different turns as previously described [31]. Due to the preparation methods, the 4.m and a turns could not always be analyzed separately. Therefore, SGN densities of these areas were averaged for analysis if available. In general, cells larger than 17 µm in diameter were counted. Depending on image quality, Rosenthal’s canal was partially covered by shadows caused by the electrode, so not all cells could be detected. The number of vital SGNs was related to the measured cross-sectional area of the Rosenthal canal to achieve the SGN density, expressed as cells/10,000 µm^2^.

### 2.10. ELISA Analysis of DEX Levels in Perilymph Samples

DEX concentration was measured using a competitive commercial enzyme-linked immunosorbent assay (ELISA) kit (Neogen Corp., Lexington, MA, USA). Due to the small sample volume, the samples from each group were pooled and filled up to 45 µL, and a double determination was performed. Artificial perilymph (145 mM NaCl (Merck KGAA), 1.2 mM CaCl_2_ (Merck KGAA), 5 mM HEPES (Merck KGAA)) was used for the negative control. A microplate reader (Tecan Trading AG, Männedorf, Switzerland) with a 650 nm filter was used to scan the plate. The sensitivity of the test was 0.23 ng/mL.

### 2.11. Statistics

Statistical evaluations were performed using GraphPad Prism 9. First, all data were tested for normal distribution using the Shapiro–Wilk test. Hearing thresholds were statistically analyzed using ANOVA. The Kruskal–Wallis test was used to compare clinical impedances between groups. In addition, unpaired *t*-tests or Mann–Whitney tests (depending on the result of the normality test) were performed for each individual day to compare clinical impedances between the individual groups. For the evaluation of connective tissue growth, each group was compared once with the control group and once with the PLLA group using the Wilcoxon test. For the evaluation of SGN density, a paired *t*-test or a Wilcoxon test was used to compare the implanted ears with the untreated ears within each group. The Kruskal–Wallis test was used for comparison between the four groups. *p*-values below 0.05 were considered to indicate significant differences.

## 3. Results

All animals were in good general condition throughout the experiment. No losses of the connectors were recorded. In four of the six guinea pigs in the diclofenac group, a white film to stalagmitic ossifications in the middle ear was observed at day 28 when the cochleae were dissected.

### 3.1. Hearing Threshold Determination

**Figure 3:**
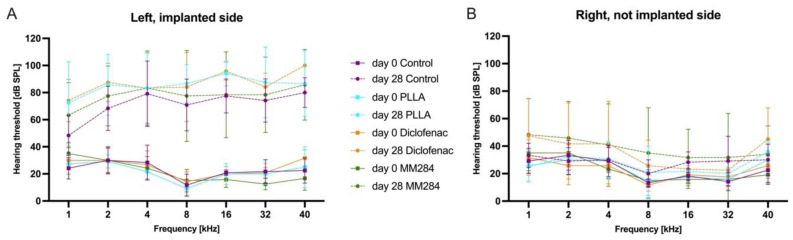
Hearing threshold determination using AABR measurement of the (**A**) left, implanted and (**B**) the right, not-implanted side on day 0 before implantation (solid lines) and after 28 days of implantation (dashed lines). Mean ± SD.

All guinea pigs were normal hearing (<50 dB SPL) at the beginning of the experiment (Figure 3) with no threshold differences between left and right ears. After 28 days, the average threshold shift on the implanted side was 48 ± 5 dB, 63 ± 4 dB, 62 ± 3 dB, and 56 ± 4 dB (mean ± SEM) in control, PLLA, diclofenac, and MM284 groups, respectively. On the control side, thresholds were increased only by 6 ± 2 dB (control), 3 ± 2 dB (PLLA), 13 ± 4 dB (diclofenac), and 15 ± 4 dB (MM284). There were no significant differences between the groups. In the last group, there was one animal with increased thresholds between 70 dB (16 kHz and 40 kHz) and 100 dB or above at 1 to 4 kHz on the non-implanted side. In general, average threshold shifts on the implanted side were between 24 dB (control) and 44 dB (PLLA and diclofenac) at 1 kHz and increased to between 59 dB (control) and 78 dB (PLLA) at 8 kHz, and 52 dB (control) and 68 dB (PLLA) at 32 dB. No differences in threshold shifts at the specific frequencies were detected between groups.

### 3.2. Clinical Impedances

**Figure 4:**
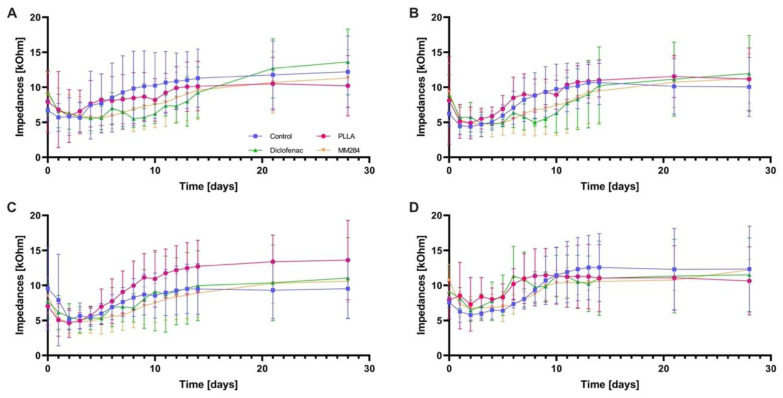
Clinical impedance measurements from day 0 to 28, divided into (**A**) contact one, (**B**) contact two, (**C**) contact three, and (**D**) contact four. Mean ± SD. Blue = Control group, pink = PLLA group, green = Diclofenac group, orange = MM284 group. Contact 4 (**D**) is closest to the cochleostomy, contact 1 (**A**) is the most apical. Significant differences are provided in Table 3 and Table 4.

On day 0, impedances were initially high (7 to 10 kOhms) but decreased until day 1 to 5.4 ± 1.5 kOhms (control), 6.4 ± 4 kOhms (PLLA), 6.7 ± 2.1 kOhms (diclofenac), and 6.2 ± 2.3 kOhms (MM284) on average over all contacts (mean ± SD). There were no differences between the groups (*p* = 0.24). Subsequently, a continuous increase was observed at all four contacts in all groups (Figure 4). The groups treated with diclofenac and immunophilin inhibitor MM284 showed a slower impedance increase in the first two weeks compared to the control and PLLA group. At day 28, impedances were 11.0 ± 4.7 kOhms in the control group, 11.4 ± 4.6 kOhms (PLLA), 12.0 ± 4.9 kOhms (diclofenac), and 11.4 ± 3 kOhms in the MM284 group, with no significant differences (*p* = 0.88). From day 0 to day 5, there were no significant differences between all groups. At contact one, there were trends toward lower impedances, especially on days 9 and 10, when comparing the diclofenac to the control group (Table 3). Between days 5 and 10, there were significantly lower impedance values at contact two in the diclofenac group compared to the control and PLLA groups (Table 4). Nevertheless, there were no significant differences after day 10. In general, no differences were observed at the more basally located contacts three and four.

### 3.3. Histology

**Figure 5:**
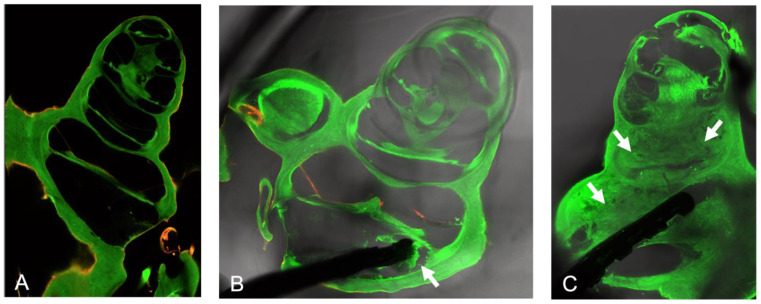
(**A**) Cochlea without implant, no connective tissue (Control group); (**B**) Cochlea with implant in the basal turn, connective tissue growth around the tip of the electrode (Control group); (**C**) Cochlea filled with connective tissue and damage caused by a coated electrode. The scala tympani of the lower basal turn is nearly free of tissue (MM284 group). Arrows = connective tissue.

Confocal laser scanning microscopy was used to evaluate the cochleae. Both connective tissue growth and SGNs were quantified from the images. Intracochlear staining using vimentin was not successful, especially when much connective tissue had formed (Figure 5C). Thus, the evaluation was performed based on autofluorescence (Figure 5). The untreated side (Figure 5A) was used as an internal group control for counting spiral ganglion neurons (Section 3.3.2). No connective tissue was detected in any right, control ears. In the implanted cochleae, the extent of connective tissue growth ranged from very little (only in the basal turn, Figure 5B) to very much (basal and middle turns filled with tissue, Figure 5C).

Increased damage was detected within the cochlea in two thirds of the animals when a coated electrode was implanted (Table 5, Figure 5C). Insertions were rated as standard implantation when the electrode array was positioned in scala tympani or as being connected to extended damage when the position of the electrode tip was in scala vestibuli or even in the second turn.

#### 3.3.1. Connective Tissue Quantification

**Figure 6:**
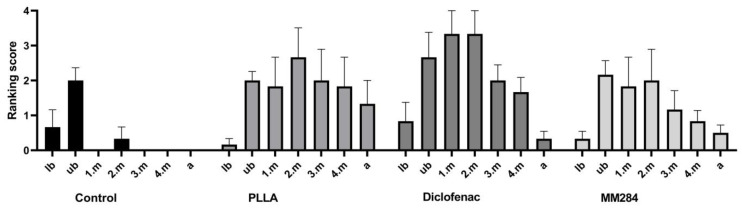
Connective tissue growth in the turns of the cochlea. lb = lower basal turn, ub = upper basal turn, 1.m = 1st middle turn, 2.m = 2nd middle turn, 3.m = 3rd middle turn, 4.m = 4th middle turn, a = apical turn. Mean ± SEM, Ranking scores according to Table 2. There were no significant differences between the PLLA group and the diclofenac and MM284 groups.

In the control group, the most connective tissue growth was in the upper basal turn (Figure 6), whereas from 3.m to the a turn, no tissue at all was found. In all groups with the coating, there was less connective tissue growth in the lower basal turns and in the apical turns than in the other turns, but all turns from base to apex could be affected by the formation of connective tissue. There was no significant difference in connective tissue growth in the individual turns of the cochlea between the PLLA group and the drug-loaded groups (diclofenac and MM284). The control group showed comparatively little connective tissue growth; here, the implant was always located in the basal turn of the cochlea. As a result, no connective tissue grew further apically, leading to the score of 0 in the ranking score. In comparison to the PLLA group, no significant differences were detected either. 

**Figure 7:**
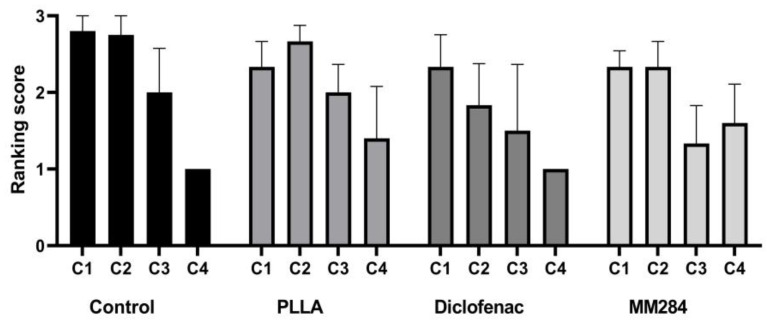
Tissue growth around the contacts, C1 = contact 1, C2 = contact 2, C3 = contact 3, C4 = contact 4, Mean ± SEM, no differences between groups were detected. Ranking score: 0 = no connective tissue, 1 = thin film of fibrosis directly on the contact, 2 = reticular growth of connective tissue on the contact, 3 = contact completely covered by compact connective tissue.

In all groups, scores for tissue growth at the position of the electrode contacts were higher for contacts one and two, which were located further apical, than for contacts three and four (Figure 7). There were no significant differences between the groups.

#### 3.3.2. Spiral Ganglion Neuron Density

**Figure 8:**
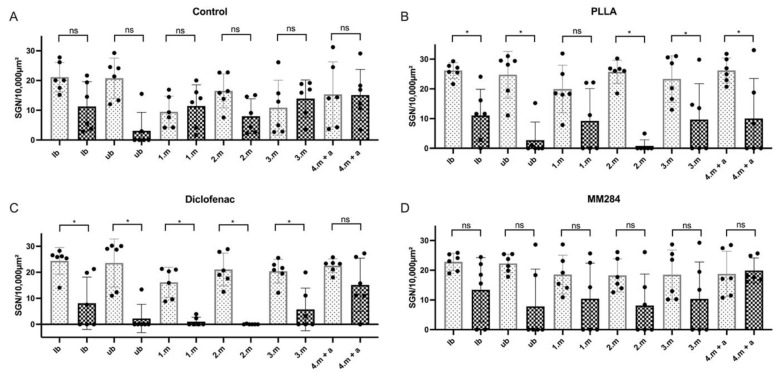
Spiral ganglion neuron density: (**A**) control group, no significant differences; (**B**) PLLA group, significant differences in all turns of the cochlea except the first middle turn; (**C**) diclofenac group, significant differences in all turns of the cochlea except the fourth middle turn; (**D**) MM284 group, no significant differences. Mean ± SD. Light gray dotted bars = right, untreated side; black checkered bars = left, implanted side. * = *p* < 0.05; ns—not significant.

For quantification of SGN survival, the untreated side was compared with the implanted side. Again, lb, ub, 1.m, 2.m, and 3.m turns were distinguished. The 4.m turn was combined with the apical turn, as here, the cell areas merged into each other. Average cell numbers for the entire cochlea were 15.7 ± 8.3 (control, mean ± SD), 24.4 ± 6.1 (PLLA), 21.4 ± 6.1 (diclofenac), and 19.9 ± 5.9 (MM284) cells per 10,000 µm³ on the untreated side. On the implanted side, these numbers were significantly reduced to 10.5 ± 7.8 (control, *p* = 0.036), 7.3 ± 9.8 (PLLA, *p* < 0.0001), 5.4 ± 8.4 (diclofenac, *p* < 0.0001), and 11.7 ± 10.8 (MM284, *p* = 0.0021) per 10,000 µm³. A more detailed presentation of the cell numbers in the different turns is provided in Figure 8. In the implanted controls, no SGNs were found in four cases in the ub turn, whereas in all other turns and cases, at least some cells survived. The numbers of turns without any SGNs increased to 19, 18, and 15 for PLLA, diclofenac, and MM284 groups, respectively. It was observed that in PLLA and diclofenac groups, values were reduced in all animals. In contrast, in the MM284 group, numbers of surviving SGNs were either zero or unchanged compared to the contralateral side. 

Differences between sides were not significant in the control group and the MM284-treated group. In contrast, in the PLLA and diclofenac groups, significantly fewer SGNs were found in the implanted side after 28 days. In the group comparison, there was a significant difference only in the first middle turn between the control group and the diclofenac group (*p* = 0.0401) and in the second middle turn between the control group and the PLLA group (*p* = 0.0348) and between the control group and the diclofenac group (*p* = 0.0229) (Kruskal–Wallis test).

### 3.4. Dexamethasone Concentration in Perilymph

**Figure 9:**
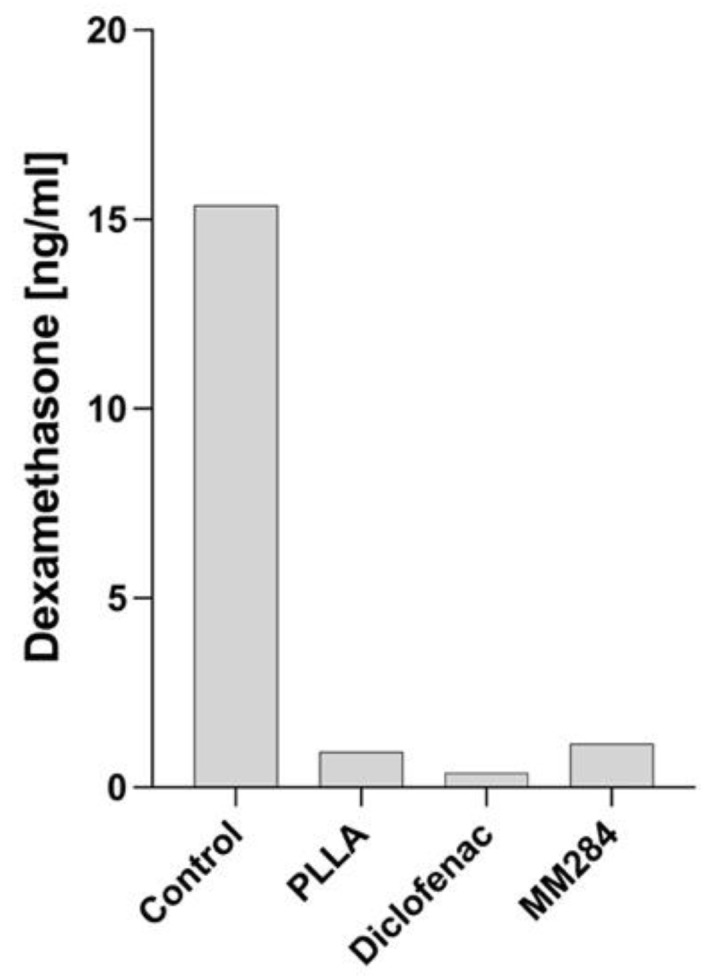
DEX concentration in perilymph. Results are from pooled samples of each group (Control, PLLA, Diclofenac, MM284). Means of double determination are shown.

An ELISA was performed to quantify the DEX release from the electrodes in the inner ear. In the control group, the highest concentration was detected in the perilymph (15.27 ng/mL) (Figure 9), followed by MM284 (1.15 ng/mL) and PLLA (0.94 ng/mL). Complementarily, in the diclofenac group, the lowest concentration was detected (0.39 ng/mL). 

## 4. Discussion

CIs have been around for decades, yet the system still presents many challenges—one of which is connective tissue growth around the electrode. This leads to poorer signal transduction and increased impedances at the electrode contacts [32,33]. Many factors affect the formation of connective tissue in the inner ear after implantation of a CI. On the one hand, the implantation procedure itself plays a major role, as one can already set significant damage during the surgery, and on the other hand, the implant leads to a foreign body reaction [34]. Local drug application in the cochlea is a possible approach to reduce connective tissue growth. In this context, a slow long-term release from the implant is desirable [13]. In addition to a potential drug, an appropriate carrier is also needed. As PLLA has already been shown to be suitable as a carrier for drugs and shows no negative effects in the inner ear [18], it was used in these experiments. The behavior of an electrode with DEX combined with a drug-eluting PLLA coating has not been previously reported.

The limitation of extended damage to PLLA-coated electrodes and the high number of damaged cochleae (Table 5) can only be explained by altered mechanical properties of the electrode array. In contrast to many positive effects of PLLA, it leads to stiffening of the electrode when used as a coating. This seems to be contradictory to the results of [19] where the generated pressure and friction during insertion of PLLA-coated electrode carriers were not increased. In the cited study, linear models of the scala tympani were used, preventing a measurable influence of altered bending characteristics of the electrode array. It is possible that the PLLA coating was too thick in combination with the electrode. In an earlier study, no increased damage was reported for PLLA-coated electrode models compared to electrode models without a coating [18]. As, in the earlier study, pure silicone models of CI electrodes (no metal parts) were used, we might speculate that the combination of an electrode array with wires and contacts and the PLLA coating leads to an increased stiffness that finally was too high for insertion in the cochlea. Therefore, in future studies, attention should be on improvement of the flexibility of the electrode. The influence of the used trauma model [2] on these observations remains unclear. As the model was also used for the control group and, in this group, no extended damage was observed, we might speculate that the observed greater trauma can be attributed to an increased stiffness of the PLLA-coated electrode arrays. However, as drug-loaded and plain PLLA coatings were used, the effects of the drugs diclofenac and immunophilin inhibitor MM284 could still be investigated. Nevertheless, the extended trauma with—at least in some cases—the tip of the electrode being located in the second turn remains disadvantageous in order to compare the study results with previous investigations using local DEX application [30]. 

Diclofenac and the immunophilin inhibitor MM284 were used in the ear for the first time in this project based on in vitro studies from Wulf et al. 2022 [21] and Goblet et al. 2022 [23]. There, the authors showed no ototoxic effects of the drugs on SGNs. Four animals from the diclofenac group showed a reaction in the middle ear that did not occur in animals from the other groups. As, to the best of our knowledge, it is the first time reporting this phenomenon, a species-dependent reaction would be the most obvious cause even though also a general effect of diclofenac in the ear cannot be excluded. However, the exact pathogenesis is not clear and requires further investigation especially as most but not all animals of this group were affected.

All animals showed a hearing loss of more than 30 dB after 28 days on the implanted ear, which can be explained by the electrode insertion trauma [35]. Increased hearing loss due to increased fibrosis has been described in the literature [36]. Ceschi et al. [18] showed lower absolute values for threshold shifts using coated and plain electrode models but, in their study, no trauma model was applied and also no extended damage was reported. Therefore, it is concluded that the larger threshold shifts in the current study are caused by the increased damage and not directly by the coating material and drugs applied. Additionally, the drilling of the cochleostomy caused intense noise, which also contributes to the hearing loss [37]. However, this study focused on the investigation of connective tissue growth. As, with a cochleostomy, more tissue growth can be expected [38], the cochleostomy approach was chosen.

Impedances obtained with the clinical system tended to be lower in the first ten days with diclofenac and MM284, especially at contacts one and two. From day 10, impedances increased to a similar level in all groups. This behavior over time indicates a delay in the impedance increase that can be attributed to diclofenac and MM284, as controls and empty PLLA-coatings exhibited a fast increase in impedance and all electrodes contained the same amount of DEX. Unfortunately, also in the current study, a long-lasting reduction in impedance was not achieved.

Confocal laser scanning microscopy was chosen for histological examinations in order to prevent loss of material due to embedding and further processing. Visualization of the material under examination proved a good performance, allowing for the study of the cochlea from all sides [12]. Despite the used trauma model, there was less connective tissue growth in the control group, as the electrode without the PLLA coating caused no extended damage. Even though a direct comparison to electrode arrays without dexamethasone is not possible, the low tissue reaction in the control group despite the application of a trauma model might also indicate a beneficial effect of the released DEX, which should be expected according to [39]. 

The concentration of DEX in the perilymph was highest in the control group. With an amount of 15.27 ng/mL, this concentration can be considered as not toxic for the SGNs [40]. In the coated electrodes, it was much lower. A PLLA coating reduces the release of DEX from the silicone [21] to less than half the value of uncoated silicone. In the current study, this difference was much larger than expected. Most likely, the amount of connective tissue contributes to this difference. In all groups with the PLLA coating, the connective tissue extended to and, in most cases, beyond the second middle turn. This tissue might have provided a diffusion barrier for the released DEX as perilymph was sampled from the apex of the cochleae. This, in turn, resulted in low volumes of collected perilymph such that all samples of a study group had to be pooled. However, most importantly, DEX was still released from the electrode array in vivo despite the PLLA coating and the tissue growth. 

Regarding the amount of connective tissue, a direct comparison between the coated electrodes and the controls remains difficult. The much larger amount of tissue in the groups with the coated electrodes can most likely be attributed to the extended damage that was caused using these electrodes. Nevertheless, an influence of the PLLA itself on fibrosis cannot completely be excluded. Only little tissue was observed on PLLA-coated electrode models in an earlier study but, in that study, a detailed analysis of the connective tissue was not performed, due to explantation of most of the electrode models [18]. Comparison of the drug-loaded groups with the PLLA group allowed an assessment on the efficacy of the drugs. No significant differences in connective tissue growth were detected, yet comparison of MM284 with diclofenac indicated less connective tissue growth in animals implanted with MM284. Thus, there was a positive trend in favor of MM284. 

This positive trend toward MM284 was also supported by the numbers of surviving SGNs. Despite the extended damage, the numbers of surviving SGNs were not different between treated and untreated sides. The density of spiral ganglion neurons of the untreated sides was, on average, between 16 SGNs/10,000 µm^2^ and 25 SGNs/10,000 µm^2^. These values match those described in the literature, which were detected by confocal laser scanning microscopy and analyzed with ImageJ as in the current study [31,41]. In contrast, with PLLA and PLLA containing diclofenac, SGN numbers were reduced when compared to the respective untreated side. As SGN numbers were not different between PLLA-coated samples and uncoated samples in [18], we speculate that the reduction in SGNs in the current study was caused by the increased trauma with coated electrodes. Then, MM284 prevented this trauma-induced reduction in SGN numbers.

## 5. Conclusions

The used PLLA coating appears to stiffen the electrode arrays too much for atraumatic insertion even though a trauma model was used. The results indicate that diclofenac and immunophilin inhibitor MM284 delay the growth of connective tissue after CI implantation. However, based on the impedances, a long-term effect is not observed. This statement is supported by the histology evaluation. It shows that on day 28, there is no significant difference in connective tissue growth around the electrode between the groups with the coated implants even though values for the MM284 group are a little lower. As SGNs appear protected by MM284, this substance might be a preferred approach for application in the cochlea compared to diclofenac. Future studies may address different coating approaches to deliver anti-inflammatory drugs into the inner ear and should, in addition, focus on relevant drug concentrations. Based on the current results, it is not expected that delaying the growth of connective tissue after cochlear implantation is sufficient for clinical application.

## Figures and Tables

**Table 1:** Overview of group division.

Group	Surface
Control	DEX-silicone
PLLA	DEX-silicone coated with PLLA
Diclofenac	DEX-silicone coated with PLLA containing diclofenac
MM284	DEX-silicone coated with PLLA containing MM284

**Table 2:** Ranking score for connective tissue growth in the entire cochlea.

Score	Connective Tissue Growth (X) [%]
0	none
1	X < 25%
2	25% ≤ X ≤ 50%
3	X > 50%
4	X = 100% (cochlear turn filled completely)

**Table 3:** *p* values contact one.

Day	Control—Diclofenac	Control—MM284	PLLA—Diclofenac	PLLA—MM284
5	0.2882	0.3169	0.147	0.1721
6	0.5072	0.2510	0.5266	0.1971
7	0.2534	0.2493	0.3085	0.3053
8	0.119	0.2719	0.2229	0.4379
9	0.0612	0.2686	0.0763	0.4942
10	0.0717	0.2742	0.179	0.7282

**Table 4:** *p* values contact two.

Day	Control—Diclofenac	Control—MM284	PLLA—Diclofenac	PLLA—MM284
5	0.1397	0.2985	0.0307 *	0.0857
6	0.6108	0.2382	0.1848	0.065
7	0.0411 *	0.305	0.0411 *	0.1532
8	0.0164 *	0.3266	0.0031 **	0.2795
9	0.0322 *	0.2902	0.0013 **	0.1901
10	0.0933	0.3394	0.0959	0.4656

* = significant, *p* < 0.05; ** *p* < 0.01.

**Table 5:** Overview of the localization of the electrode in the cochlea after implantation.

Group	Standard Implantation	Extended Damage
Control	6	0
PLLA	2	4
Diclofenac	2	4
MM284	2	4

Extended damage = implantation of the electrode in scala vestibuli or even in the middle turns.

## Data Availability

All the data that support the findings of this study are available on request from the corresponding author.
